# Cerebral Cortex Communications Inaugural Editorial

**DOI:** 10.1093/texcom/tgaa001

**Published:** 2020-02-11

**Authors:** Pasko Rakic

**Affiliations:** Cerebral Cortex Communications

We are pleased to introduce *Cerebral Cortex Communications*, a new sister journal to *Cerebral Cortex*. Unlike its parent monthly journal, the new journal, *Cerebral Cortex Communications*, does not have size constraints. It is an online-only, fully open access, rapid publication journal published by Oxford University Press. Accordingly, the journal is designed to be fully compliant with Plan S, the open science publishing initiative, as it is currently outlined. With fully open access journal papers that are available worldwide immediately after acceptance, we expect an increase in the size of the readership as well as in the ability to be cited. These days, many funders require publication in fully open access journals, and *Cerebral Cortex Communications* accommodates that requirement.

Like its parent journal, *Cerebral Cortex Communications* is dedicated to publishing high quality, rigorous articles about the development, organization, plasticity, and function of the cerebral cortex, including the hippocampus as well as the thalamocortical relationship and cortico-subcortical interactions. The journal is multidisciplinary and welcomes studies that address major issues of general significance using the most advanced neurobiological and neuropsychological techniques, including neuroanatomy, biochemistry, molecular neurobiology, genetics, electrophysiology, behavior, theoretical modeling, and brain imaging and/or electroencephalographic studies on developing and adult laboratory animal models and humans. We are also interested in cortical disorders, including autism spectrum disorder (ASD), attention-deficit hyperactivity disorder (ADHD), Schizophrenia, Alzheimer’s Disease, and aging. To ensure the same level of editorial quality, the present Editor-in-Chief and Reviewing Editors will serve on both journals, but in order to accommodate direct submissions to the new journal, it will also have several additional Associate Editors.

*Cerebral Cortex Communications* does not accept presubmission inquiries. It has two sources of submissions. The first source is the manuscripts that the authors submit directly to *Cerebral Cortex Communications.* The second source of papers includes those that were submitted to the parent journal but are deemed to be more appropriate for *Cerebral Cortex Communications*. Such papers can be, with approval of the authors, transferred to *Cerebral Cortex Communications* and, upon positive response to the reviewers’ concerns, published online.

*Cerebral Cortex Communications* will comply with a rapid publication model, and uncorrected proofs of accepted manuscripts will be quickly and freely available on Advance Access. Once proofs are corrected, the edited versions will be published online. Articles whose funders require indexing in PubMed and PubMed Central will be deposited there, and PMCID numbers will be assigned.

In conclusion, we believe that *Cerebral Cortex Communications* provides an opportunity to accept and publish more excellent papers in this exciting and rapidly growing field.

*Pasko Rakic MD, PhD.*

*Editor-in-Chief*

*Cerebral Cortex*

*Cerebral Cortex Communications*

